# Zofenopril Protects Against Myocardial Ischemia–Reperfusion Injury by Increasing Nitric Oxide and Hydrogen Sulfide Bioavailability

**DOI:** 10.1161/JAHA.116.003531

**Published:** 2016-07-05

**Authors:** Erminia Donnarumma, Murtuza J. Ali, Amanda M. Rushing, Amy L. Scarborough, Jessica M. Bradley, Chelsea L. Organ, Kazi N. Islam, David J. Polhemus, Stefano Evangelista, Giuseppe Cirino, J. Stephen Jenkins, Rajan A. G. Patel, David J. Lefer, Traci T. Goodchild

**Affiliations:** ^1^ Cardiovascular Center of ExcellenceLouisiana State University Health Sciences CenterNew OrleansLA; ^2^ Department of CardiologyLouisiana State University Health Sciences CenterNew OrleansLA; ^3^ Menarini Ricerche S.p.a., Preclinical DevelopmentFlorenceItaly; ^4^ Department of PharmacyUniversity of Naples “Federico II”NaplesItaly; ^5^ Ochsner Medical CenterNew OrleansLA

**Keywords:** antihypertensive agent, hydrogen sulfide, myocardial ischemia, nitric oxide, oxidant stress, troponin, Ischemia, ACE/Angiotension Receptors/Renin Angiotensin System, Endothelium/Vascular Type/Nitric Oxide, Oxidant Stress, Biomarkers

## Abstract

**Background:**

Zofenopril, a sulfhydrylated angiotensin‐converting enzyme inhibitor (ACEI), reduces mortality and morbidity in infarcted patients to a greater extent than do other ACEIs. Zofenopril is a unique ACEI that has been shown to increase hydrogen sulfide (H_2_S) bioavailability and nitric oxide (NO) levels via bradykinin‐dependent signaling. Both H_2_S and NO exert cytoprotective and antioxidant effects. We examined zofenopril effects on H_2_S and NO bioavailability and cardiac damage in murine and swine models of myocardial ischemia/reperfusion (I/R) injury.

**Methods and Results:**

Zofenopril (10 mg/kg PO) was administered for 1, 8, and 24 hours to establish optimal dosing in mice. Myocardial and plasma H_2_S and NO levels were measured along with the levels of H_2_S and NO enzymes (cystathionine β‐synthase, cystathionine γ‐lyase, 3‐mercaptopyruvate sulfur transferase, and endothelial nitric oxide synthase). Mice received 8 hours of zofenopril or vehicle pretreatment followed by 45 minutes of ischemia and 24 hours of reperfusion. Pigs received placebo or zofenopril (30 mg/daily orally) 7 days before 75 minutes of ischemia and 48 hours of reperfusion. Zofenopril significantly augmented both plasma and myocardial H_2_S and NO levels in mice and plasma H_2_S (sulfane sulfur) in pigs. Cystathionine β‐synthase, cystathionine γ‐lyase, 3‐mercaptopyruvate sulfur transferase, and total endothelial nitric oxide synthase levels were unaltered, while phospho‐endothelial nitric oxide synthase^1177^ was significantly increased in mice. Pretreatment with zofenopril significantly reduced myocardial infarct size and cardiac troponin I levels after I/R injury in both mice and swine. Zofenopril also significantly preserved ischemic zone endocardial blood flow at reperfusion in pigs after I/R.

**Conclusions:**

Zofenopril‐mediated cardioprotection during I/R is associated with an increase in H_2_S and NO signaling.

## Introduction

Angiotensin‐converting enzyme inhibitors (ACEIs), initially approved for the treatment of hypertension, are now widely used to improve the clinical prognosis of patients following acute myocardial infarction (AMI) and in those with congestive heart failure; however, to date, the precise mechanism(s) of their beneficial actions remain(s) poorly understood. Activation of the renin‐angiotensin‐aldosterone system (RAAS) is generally thought to be the primary pathway implicated in the pathogenesis of AMI, and its blockade by ACEI has proved to be useful in preventing subsequent cardiovascular events in patients after AMI.^1^, ^2^, ^3^, ^4^ Hansen et al^5^ examined the efficacy of different ACEIs after AMI, revealing no differences in the risk of mortality and reinfarction among all ACEIs, suggesting a class effect. In contrast, the Survival of Myocardial Infarction Long‐Term Evaluation (SMILE) clinical trial, which enrolled >3600 patients with coronary heart disease, revealed that early AMI treatment with zofenopril, an ACEI containing a sulfhydryl group, reduces mortality and morbidity to a greater extent than does ramipril, a dicarboxylate ACEI.^6^, ^7^, ^8^, ^9^ In addition to contradictions with regard to whether there is a selective superiority of certain ACEIs or only a class effect among all the ACEIs, there appears to be conflicting evidence of the anti‐ischemic effects of ACEIs, with some having shown efficacy for all ACEIs,^10^ some failing to observe any anti‐ischemic actions^11^ and still others having observed beneficial effects only for sulfhydryl‐containing agents.^12^ Within the studies showing positive cardiovascular benefit with ACEIs, there is no consensus as to the mechanism of action by which ACEIs confer cardioprotection. In isolated heart models, the anti‐ischemic actions of ACEIs have been attributed to multiple pathways, including inhibition of cardiac angiotensin II formation,^13^ reduction in bradykinin (BK) degradation resulting in enhanced nitric oxide (NO) bioavailability,^14^ oxygen radical scavenging,^15^ and altered prostaglandin production.^16^ Zofenopril is a sulfhydryl ACEI characterized by high lipophilicity, long‐lasting tissue penetration, selective inhibition of cardiac ACE, potent antioxidant activities, and effectiveness observed after single daily administration.^17^ In both preclinical^18^, ^19^ and clinical studies,^20^, ^21^ zofenopril has been shown to exert vasculoprotective and cardioprotective actions independent of its potent effects of blood pressure lowering via blockade of the RAAS.^22^ While zofenopril has been shown to reduce cardiovascular morbidity and mortality in patients with left ventricular hypertrophy and post myocardial infarction, the mechanisms by which zofenopril protects the ischemic and failing myocardium have not been fully elucidated.

Recently, experimental evidence has suggested the involvement of hydrogen sulfide (H_2_S) as yet another mechanism by which zofenopril improves peripheral vascular function independent of its ability to inhibit ACE.^23^ H_2_S is a cytoprotective physiological signaling molecule that acts in concert with NO and carbon monoxide (CO) to maintain physiological homeostasis in both the heart and circulation. H_2_S is produced in mammalian tissue by 3 tissue‐specific enzymes: cystathionine γ‐lyase (CSE), cystathionine β‐synthase (CBS), and 3‐mercaptopyruvate sulfur transferase (3‐MST).^24^, ^25^ Recent experimental evidence has shown that H_2_S is a potent cardioprotective signaling molecule, and the administration of H_2_S donors significantly attenuates the pathological consequences of myocardial ischemia/reperfusion injury (I/R)^26^ and heart failure.^27^ Indeed, it has been demonstrated that a bolus injection of an H_2_S donor, either before ischemia or at time of reperfusion, markedly ameliorates I/R injury.^26^ Similarly, cardiac overexpression of CSE protects against acute I/R injury by attenuating oxidative stress, inhibiting apoptosis, and reducing inflammation.^26^, ^28^ As an antioxidant, H_2_S has been shown to (1) directly reduce superoxide anion ($O_{2}^{-}$)^29^, ^30^ and other toxic free radical species like peroxynitrite^31^; (2) increase intracellular glutathione (GSH) synthesis^32^ and thioredoxin (Trx‐1) levels^33^; (3) promote cellular antioxidant gene expression such as glutathione peroxidase (GPX) and heme oxygenase 1 (HO‐1) that detoxify pro‐oxidative stressors; and (4) inhibit mitochondrial reactive oxygen species (ROS) production via p66Shc‐dependent signal transduction.^34^

In addition to the cardioprotective effects exerted by H_2_S alone, there are a number of studies demonstrating cross‐talk between H_2_S and NO signaling pathways,^35^, ^36^, ^37^ further enhancing cardiovascular benefit. The biological profiles of H_2_S and NO are similar as both molecules are known to protect cells against various injuries along with modulation of cellular metabolism and both are important regulators of vessel tone, oxidative stress, and apoptosis. Evidence for cross‐talk between H_2_S and NO includes improvement in survival after cardiac arrest and cardiopulmonary resuscitation by H_2_S in an endothelial nitric oxide synthase (eNOS)‐dependent manner.^38^ An additional mechanism by which H_2_S confers cardioprotection against I/R injury and pressure‐overload heart failure is through its ability to enhance eNOS activity and thereby increase myocardial NO bioavailability.^39^, ^40^

Taking into account the positive biological effects H_2_S has on the cardiovascular system and zofenopril's selective accumulation within cardiac tissue, we hypothesized that zofenopril would attenuate I/R injury though H_2_S‐ and NO‐dependent and ‐independent mechanisms. By increasing cardiac tissue and circulating plasma levels of H_2_S, we hypothesized that cardioprotection after zofenopril treatment would occur via H_2_S alone and through cross‐talk–mediated NO signaling to limit myocardial cell death. In the present study, we examined the effects of a single dose or prolonged pretreatment with zofenopril on H_2_S and NO bioavailability as well as infarct (INF) size using in vivo murine and swine models of I/R injury.

## Materials and Methods

### Animals

Male C57BL/6J mice were purchased from the Jackson Laboratory and were 10 to 14 weeks of age at the time of the experiments. All animals were housed in a temperature controlled animal facility with 12 hour light/dark cycle, water, and rodent chow provided ad libidum. Female Yucatan miniswine obtained from S&S Farms weighed 40 to 45 kg and were 9 to 10 months of age at the time of the experimental procedures. Pigs were acclimated and maintained on standard commercial diet (Teklad Miniswine Diet 8753; Harlan Laboratories). All the experimental protocols were approved by the Institute for Animal Care and Use Committee at Louisiana State University Health Sciences Center and handled in compliance with the National Institutes of Health “Guide for the Care and Use of Laboratory Animals”. Institutional review board approval was obtained.

### Treatment

#### Murine study

Mice were administered a single dose of vehicle (carboxymethylcellulose 0.2% m/v; Santa Cruz), zofenopril calcium {[(1(*S*),4(*S*)]‐1(3‐mercapto‐2 methyl‐1‐oxopropyl)4‐phenyl‐thio‐l‐proline‐*S*‐benzoylester, 10 mg/kg; Menarini Ricerche S.p.A., Italy}, or ramipril (3 mg/kg; Abcam) per oral gavage. After treatment, mice were killed at 1, 8, or 24 hours to collect heart tissue and plasma for subsequent molecular and biochemical studies or subjected to the surgical protocol for in vivo myocardial I/R.

#### Swine study

Pigs were randomly assigned to receive placebo or zofenopril calcium (Bifril, 30 mg/daily PO; Menarini Manufacturing Logistics and Service s.r.l., Italy). Treatment was started 1 week before (day −7) the I/R procedure and continued for 2 days during reperfusion. Plasma samples after 1 week of placebo or zofenopril treatment (day 0) were obtained after general anesthesia just before myocardial ischemia induction in order to measure circulating biomarkers H_2_S, nitrite (${\text{NO}}_{2}^{-}$), sulfane sulfur, and *S*‐nitrosothiol (RXNO) levels. Serial plasma samples during I/R procedure were obtained to measure circulating cardiac troponin I (cTn‐I) levels.

### Measurement of H_2_S and Sulfane Sulfur Levels

H_2_S levels were measured in plasma and heart tissue samples obtained from mice treated with vehicle, zofenopril, or ramipril at 1, 8, or 24 hours. H_2_S and sulfane sulfur levels were measured in pig plasma after 1 week of placebo or zofenopril treatment. H_2_S and sulfane sulfur levels were determined by using gas chromatography coupled with sulfur chemiluminescence according to previously described methods.^28^

### Measurement of NO Metabolites

${\text{NO}}_{2}^{-}$ levels in plasma and heart tissue obtained from mice treated with vehicle, zofenopril, or ramipril at 8 hours and in plasma obtained from miniswine following 1 week of placebo or zofenopril treatment were quantified by using HPLC methods as described previously.^41^ RXNO levels in pig plasma following 1 week of placebo or zofenopril treatment were measured by chemiluminescence detection as previously reported.^39^

### Reverse Transcription–Quantitative Polymerase Chain Reaction

Mouse myocardial RNA was isolated after 8 hours of vehicle or zofenopril treatment with the use of TRIzol reagent. Reverse transcription was performed in a standard fashion with iScript cDNA synthesis kit (BioRad). TaqMan quantitative polymerase chain reaction was carried out according to the manufacturer's instructions by using probe sets for CBS, CSE, and 3‐MST. Data analysis was carried out using *18s* as the housekeeping gene and expressed as 2^ΔΔ^CT.

### Western Blot

Whole cell lysates were prepared using myocardial tissue obtained from mice treated with vehicle or zofenopril at 8 hours. Equal amounts of protein (30 μg), determined by using the BSA Protein Assay, were separated on Tris‐HCl gel (4–20%, Bio‐Rad) and transferred to nitrocellulose membranes.^42^ The membranes were blocked and then probed with the following primary antibodies overnight at 4°C: CBS (Santa Cruz), CSE (CTH; Abnova), 3‐MST (Novus), eNOS (BD Biosciences), phospho‐eNOS 1177 (p‐eNOS^1177^; Abcam), phospho‐eNOS 495 (p‐eNOS^Thr495^; Cell Signaling Technology), glutathione peroxidase 1 (GPx‐1; Santa Cruz), thioredoxin 1 (Trx‐1; Santa Cruz), Cu,Zn‐superoxide dismutase (SOD‐1; Santa Cruz), and glyceraldehyde‐3‐phosphate dehydrogenase (GAPDH; Santa Cruz). Immunoblots were probed with the appropriate fluorescence conjugate secondary antibodies for 2 hours at room temperature and visualized with the Odyssey Imaging System (LI‐COR Biotechnology), then quantified by using Image J software.

### Murine In Vivo Myocardial I/R Protocol

The surgical protocol for in vivo I/R was performed similar to methods as described previously.^39^ Briefly, mice were pretreated with vehicle, zofenopril, or ramipril 8 hours before I/R. Mice were anesthetized with ketamine (60 mg/kg IP) and pentobarbital (50 mg/kg IP), ventilated, and placed on a heating table maintained at 37°C. After surgical thoracotomy, myocardial ischemia was induced by occlusion of the left coronary artery using 7‐0 silk suture and 3 to 5 mm of PE‐10 tubing. After 45 minutes of occlusion, the suture and tubing were removed, the surgical site was closed, and the mice were treated with buprenorphine (0.1 mg/kg) and cefazolin (60 mg/kg), recovered, and reperfused for 24 hours.

### Swine Model of Myocardial I/R Protocol

I/R was performed similar to as previously described.^43^ Pigs were randomized to receive placebo (n=8) or zofenopril (30 mg/daily PO; n=9) for 7 days before the I/R procedure and 2 days during reperfusion and received aspirin (81 mg PO) 1 day before the I/R procedure. Pigs were sedated with ketamine:xylazine (15:1 mg/kg IM), administered diazepam (0.5 mg/kg IV) to aid with intubation, mechanically ventilated, and anesthetized by using methohexital (Brevital sodium 7.0–8.0 mg/kg per hour IV). Pigs received aspirin (300 mg IV) and antibiotic (ceftiofur sodium [Naxcel] 3 mg/kg IM), ECG, heart rate, respiration, O_2_ saturation, arterial blood pressure, and body temperature were continuously monitored. With the use of standard sterile technique, appropriately sized sheaths were placed via cutdown in the right and left femoral arteries for placement under fluoroscopic guidance (Optima CL323i; GE) of a 6F hockey stick catheter (Cordis) for angiography and balloon catheter placement and a 5F pigtail catheter (Cordis) for left ventricular (LV) microsphere injections. Heparin (300 U/kg IV) was administered, and activated clotting time maintained >250 seconds. Myocardial ischemia was generated by 75 minutes of angioplasty balloon occlusion (2.5–3.0×6 mm, EMPIRA RX PTCA; Cordis) of the proximal left anterior descending coronary artery (LAD) followed by balloon deflation and reestablishment of blood flow. A 7F indwelling right jugular venous catheter (Hickman; Bard) was placed via cutdown for serial blood draws. Buprenorphine (0.025 mg/kg IM) analgesia was administered and the pigs recovered. After 48 hours of reperfusion, pigs were sedated, ventilated as described earlier, anesthetized with isoflurane (1–3% in O_2_), and administered heparin (300 U/kg IV), and a right carotid cutdown was performed for LV catheter microsphere injections as described earlier. Pigs were then killed with potassium chloride (40 mEq/kg IV). The heart was harvested for INF size assessment.

### Measurement of Infarct Size

Mouse hearts were perfused in situ with Evans blue buffer (7%), excised, sliced into 5 sections, and incubated in 1% 2,3,5‐triphenyltetrazolium chloride buffer for 3 minutes at 37°C. Pig hearts were mounted onto a dual perfusion system and maintained at ≈80 mm Hg at 37°C. The LAD was cannulated at the previous in vivo occlusion site to perfuse the ischemic/reperfused myocardium (area‐at‐risk [AAR]) with 1% 2,3,5‐triphenyltetrazolium chloride buffer and the aortic root for retrograde perfusion with 5% solution of Phthalo blue to delineate the INF and nonischemic regions, respectively. The heart was then sectioned from apex to base into 9 or 10 transverse slices. All mouse and pig heart slices were analyzed for determination of AAR/LV, INF/AAR, and INF/LV by using Image J software.

### Measurement of Regional Myocardial Blood Flow

Regional myocardial blood flow (RMBF) was measured in pig myocardial tissue and blood samples by using stable‐isotope neutron‐activated microspheres (BioPhysics Assay Laboratory, Inc) as described previously.^44^ Microspheres were injected at 4 procedural time points: baseline (time 0 minute ischemia), 60 minutes of ischemia, 15 minutes after reperfusion and 48 hours after reperfusion. At each time point, a total of 5×10^6^ microspheres (2 mL) labeled with samarium, europium, lutetium, or lanthanum was injected into the LV cavity through the pigtail catheter. A reference blood sample was drawn from the side arm of the arterial sheath catheter by using a withdrawal pump at 7 mL/min for 90 seconds. Transmural LV blocks (≈1 g) were obtained from both the ischemic zone and nonischemic zone and divided into endocardial and epicardial halves. Tissue and blood samples were processed according to manufacturers' instructions and sent for analysis. Absolute RMBF (mL/min per gram) was calculated by using the following formula:

$$\begin{array}{cc} \text{RMBF}= & (\text{counts in tissue sample}\times \text{reference blood}\\ & \text{sample withdrawal rate})/(\text{counts in the}\\ & \text{reference blood sample}\times \text{tissue weight in grams}) \end{array}$$

### Measurement of cTn‐I Release

Although absolute cTn‐I elevations are seen in multiple chronic cardiac and noncardiac conditions, a rise or fall in serial cTn‐I levels strongly supports an acutely evolving cardiac injury, most commonly, AMI.^45^ For both species, blood was collected in heparinized tubes and centrifuged at 1500 *g* for 15 minutes at 4°C to separate plasma. For mice, a ≈50 μL blood sample was obtained from the tail vein at 4 hours of reperfusion. Plasma was used to measure circulating cTn‐I as an additional index of cardiac injury by using a high sensitivity mouse‐specific ELISA kit (Life Diagnostics). For pigs, serial blood samples (≈4.0 mL each) were collected at baseline (day 0), 60 minutes of ischemia, and after 15 minutes as well as after 2, 4, 6, 24, and 48 hours of reperfusion. Plasma cTn‐I release was determined by using a high sensitivity porcine‐specific ELISA kit according to the manufacturer's instructions (Life Diagnostics).

### Statistical Analysis

All data presented in this study are expressed as mean±SEM. Differences between the groups were compared by using Prism 6 (GraphPad Software). Statistical analysis was determined by using Student unpaired, 2‐tailed *t* test or ANOVA followed by Dunnett as a post hoc test. Differences were considered statistically significant when the *P* value was <0.05.

## Results

### Zofenopril Increases H_2_S Bioavailability In Vivo

**Figure 1:**
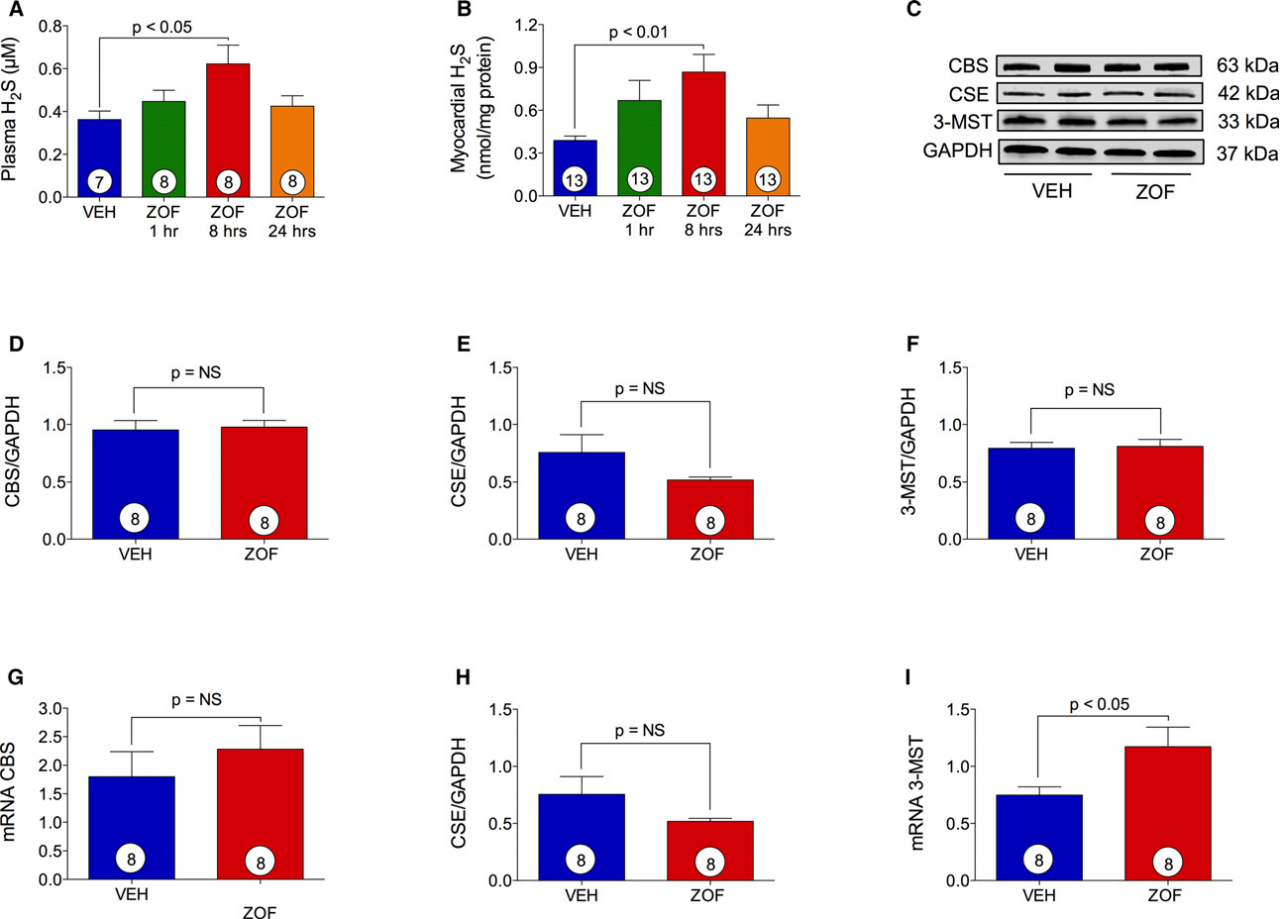
Zofenopril increases H_2_S bioavailability. VEH indicates vehicle; ZOF, zofenopril. Effect of vehicle and zofenopril treatment (10 mg/kg PO) at 1, 8, or 24 hours on H_2_S levels in mice plasma (A) and heart tissue (B). Zofenopril administration induced a significant increase in circulating and cardiac H_2_S levels at 8 hours of treatment as compared with vehicle. C, Immunoblots for cystathionine γ‐lyase (CSE), cystathionine β‐synthase (CBS), and 3‐mercaptopyruvate sulfur transferase (3‐MST) with relative optical densitometry (D through F). 8 hours of zofenopril treatment did not cause any change in CBS (D), CSE (E), and 3‐MST (F) protein expression. G through I, mRNA level of H_2_S‐producing enzymes after zofenopril therapy for 8 hours. Zofenopril treatment did not affect CBS (G) or CSE (H) gene expression, but it induced a significant increase in 3‐MST (I) mRNA levels compared with vehicle. Results are expressed as mean±SEM. Number in the circle inside the bar denotes the number of animals used per each group.

To determine whether zofenopril increases circulating and tissue H_2_S bioavailability, mice received a single administration of vehicle or zofenopril (10 mg/kg) and were killed at 1, 8, or 24 hours. As seen in Figure 1A and 1B, administration of zofenopril to mice resulted in a significant increase in H_2_S bioavailability in both plasma (0.62±0.09  versus 0.36±0.04 μmol/L, *P*<0.05) and myocardium (0.87±0.12  versus 0.39±0.03 nmol/mg protein, *P*<0.01) at 8 hours after treatment compared with vehicle. Plasma and myocardial tissue H_2_S levels were similar to vehicle at both 1 and 24 hour time points. Therefore, all subsequent experiments investigating zofenopril's cardioprotective effects in mice were performed at 8 hours of treatment for maximal H_2_S bioavailability. To determine whether zofenopril's effect on H_2_S levels is dose dependent, a lower dose of zofenopril (6 mg/kg) was administered. At 8 hours after treatment, myocardial tissue H_2_S levels were similar to vehicle (data not shown), suggesting that zofenopril modulates myocardial tissue levels of H_2_S levels in a dose‐dependent manner. We next determined whether elevations in myocardial H_2_S levels resulted from H_2_S release by zofenopril or whether zofenopril treatment led to increased expression of H_2_S‐producing enzymes CBS, CSE, and 3‐MST. Although there was no change in protein expression of CBS, CSE, and 3‐MST (Figure 1C through 1F), zofenopril treatment led to a significant increase in mRNA expression of cardiac 3‐MST (Figure 1I) compared with vehicle (1.17±0.17 versus 0.75±0.07, *P*<0.05). These findings suggest that the elevations in tissue and plasma H_2_S levels we observed are primarily because of zofenopril's ability to act as an H_2_S donor in vivo and not because of increased expression of H_2_S‐generating enzymes. Thus, the observed increase in H_2_S bioavailability is likely directly attributed to H_2_S release in vivo from zofenopril.^23^

### Zofenopril Activates eNOS and Increases NO Bioavailability

**Figure 2:**
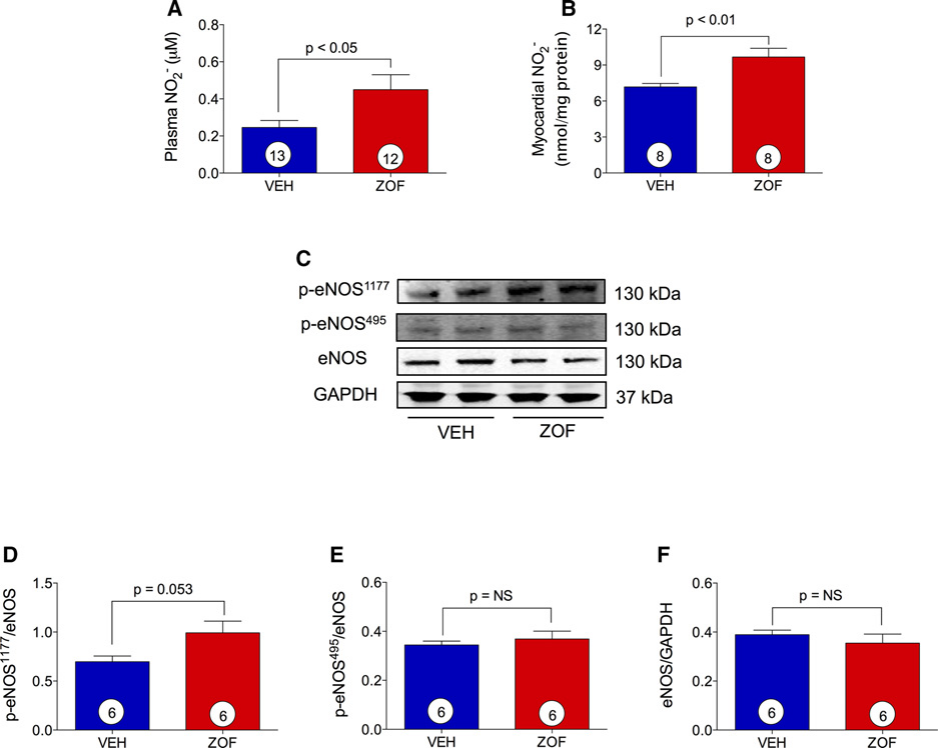
Zofenopril effect on NO bioavailability and myocardial p‐eNOS ^1177^, p‐eNOS ^495^, and eNOS expression. VEH indicates vehicle; ZOF, zofenopril. Zofenopril administered at 8 hours (10 mg/kg PO) resulted in significant increase of nitrite levels in both (A) plasma and (B) heart tissue compared with vehicle. C, Immunoblots for p‐eNOS ^1177^, p‐eNOS ^495^, and eNOS with relative optical densitometry (D through F). Results are expressed as mean±SEM. Number inside the circles in the bar denotes the number of animals used per group.

Since inhibition of ACE and H_2_S when administered independently is known to increase NO levels, we sought to determine whether the combination of ACEI and H_2_S release by zofenopril further augmented NO bioavailability. Plasma samples and myocardial tissue were obtained from healthy mice treated with vehicle or zofenopril at 8 hours and analyzed for NO metabolites. As shown in Figure 2A and 2B, there was a significant increase in both plasma and myocardial tissue ${\text{NO}}_{2}^{-}$ levels in zofenopril‐treated animals compared wih vehicle (0.45±0.08  versus 0.25±0.04 μmol/L, *P*<0.05; 9.67±0.73  versus 7.18±0.29 nmol/mg protein, *P*<0.01, respectively). We then determined the effects of zofenopril on myocardial eNOS expression, as well as phosphorylation status at the activation site p‐eNOS^1177^ (Ser1177) and at the inhibitory site p‐eNOS^495^ (Thr495) (Figure 2C through 2F). Zofenopril administration at 8 hours increased p‐eNOS^1177^ (*P*=0.053), but this difference did not achieve statistical significance compared with vehicle (Figure 2D). We observed that p‐eNOS^495^ and total eNOS expression were similar between zofenopril‐ and vehicle‐treated mice (Figure 2E and 2F). Taken together, a single administration of zofenopril in healthy mice increased myocardial and circulating ${\text{NO}}_{2}^{-}$ levels and a trend in increased activation of eNOS.

### Ramipril, a Dicarboxylate ACEI, Does Not Affect H_2_S and NO Bioavailability

**Figure 3:**
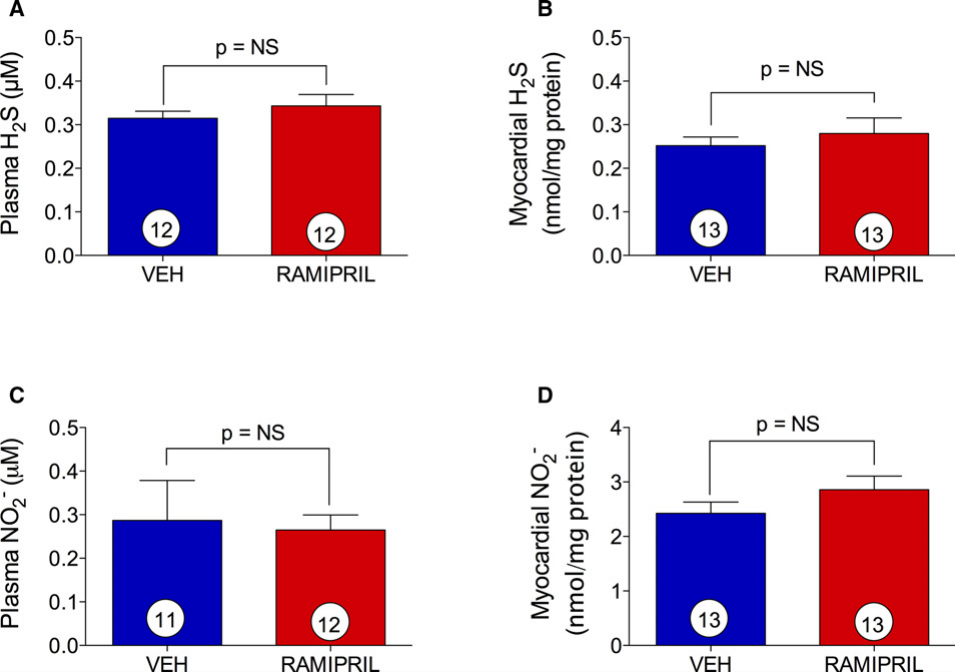
Ramipril effect on H_2_S and NO bioavailability. H_2_S and NO determination in mice plasma (A and C) and myocardial tissue (B and D) after 8 hours of vehicle (VEH) or ramipril (3 mg/kg PO) treatment. Ramipril did not affect basal levels of H_2_S and NO. Results are expressed as mean±SEM. Number in the circle inside the bar denotes the number of animals used per group. NO indicates nitric oxide.

To determine whether ACE inhibition itself leads to increased myocardial tissue and circulating H_2_S levels, ramipril, a non‐sulfhydryl ACEI, was administered (3 mg/kg) at 8 hours before the mice were killed. It has been shown that ramipril is 3‐fold more potent on a milligram basis with respect to RAAS inhibition compared with zofenopril and, therefore, the 3 mg/kg dose was chosen so as to maintain consistency of RAAS inhibition between zofenopril and ramipril treatment groups.^46^ After ramipril administration, H_2_S levels in plasma (0.34±0.03  versus 0.32±0.02 μmol/L, *P*=0.196; Figure 3A) and heart tissue (0.28±0.04  versus 0.25±0.02 nmol/mg protein, *P*=0.499; Figure 3B) were similar compared with vehicle. Similarly, there was no effect of ramipril on ${\text{NO}}_{2}^{-}$ levels detected 8 hours after treatment in both plasma (0.27±0.04  versus 0.29±0.09 μmol/L, *P*=0.831; Figure 3C) and myocardial tissue (2.86±0.25  versus 2.42±0.21 nmol/mg protein; Figure 3D).

### Zofenopril Upregulation of Myocardial Antioxidant Enzymes

**Figure 4:**
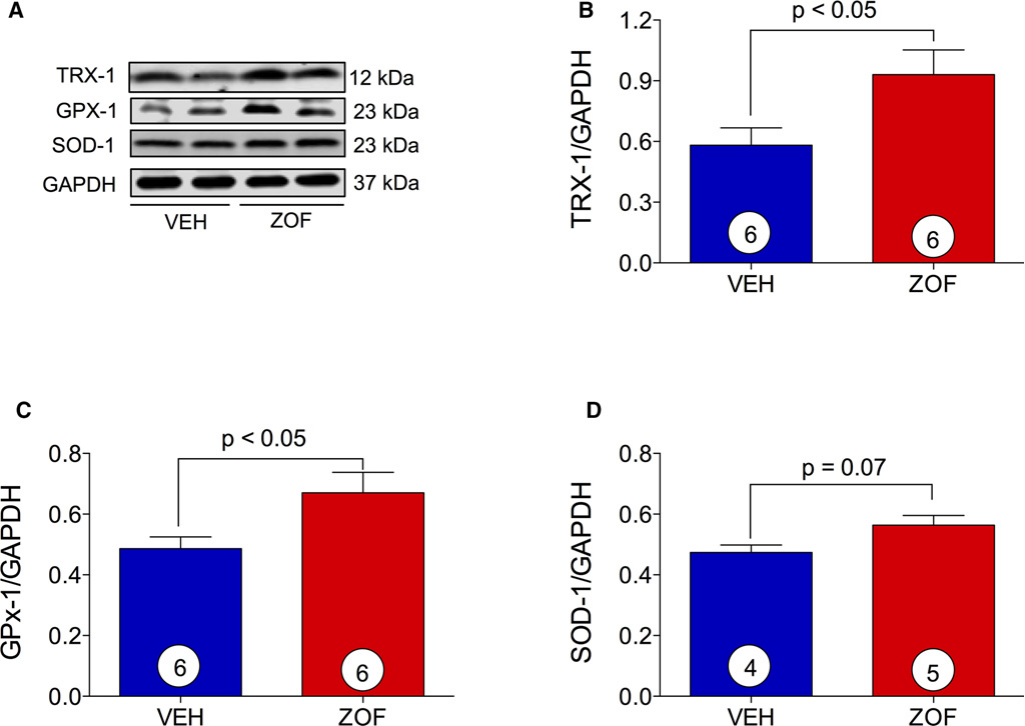
Induction of myocardial antioxidant protein. VEH indicates vehicle; ZOF, zofenopril. A, Immunoblots for Trx‐1, GPx‐1, and SOD‐1 with relative optical densitometry (B through D). Zofenopril administration promoted a significant upregulation of antioxidant proteins Trx‐1 (B) and GPx‐1 (C) and an induction of SOD‐1 (D). Results are expressed as mean±SEM. Number in the circle inside the bar denotes the number of animals used per group.

The potential for zofenopril to modulate antioxidant protein expression in myocardial tissue was determined for Trx‐1, GPx‐1, and SOD‐1 (Figure 4A through 4D). Zofenopril administration significantly upregulated Trx‐1 and GPx‐1 protein expression compared with vehicle (Figure 4B and 4C; *P*<0.05). SOD‐1 protein levels trended higher within the myocardium after zofenopril treatment compared with vehicle, although they did not reach statistical significance (Figure 4D).

### Zofenopril Protects Against Acute Myocardial I/R Injury in Mice

**Figure 5:**
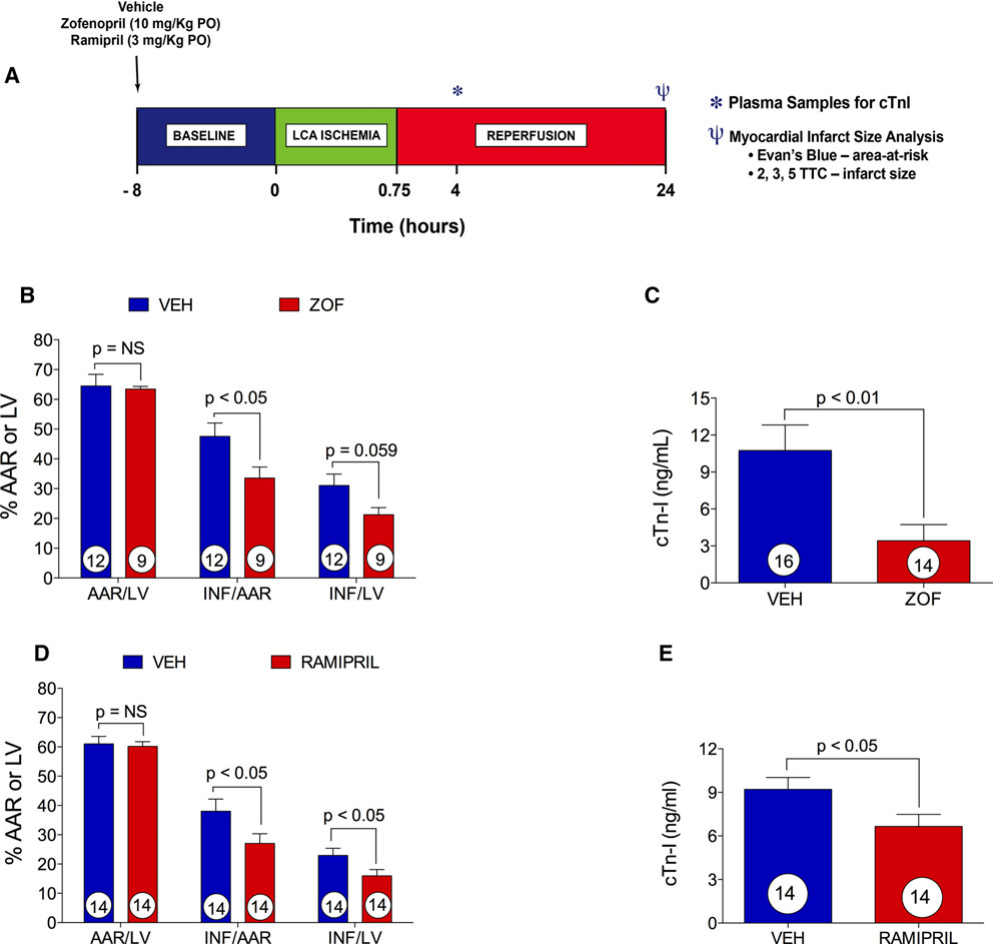
Zofenopril and ramipril effect on a murine model of myocardial ischemia reperfusion (I/R) injury. VEH indicates vehicle; ZOF, zofenopril. A, Murine model of myocardial I/R protocol. B, Area‐at‐risk (AAR) per left ventricle (LV) and infarct (INF) per AAR and LV. Zofenopril administration (10 mg/kg PO) 8 hours before induction of myocardial I/R injury significantly reduced INF/AAR compared with vehicle. There was no difference in AAR/LV between vehicle‐ and zofenopril‐treated animals. C, Circulating cTn‐I levels measured at 4 hours of reperfusion were significantly diminished by zofenopril pretreatment. D, Ramipril administration (3 mg/kg PO) 8 hours before induction of I/R injury significantly reduced the % INF/AAR and % INF/LV. E, cTn‐I release at 4 hours of reperfusion was significantly reduced by ramipril administration compared with vehicle. Results are expressed as mean±SEM. Number in the circle inside the bar denotes the number of animals used per group.

We next investigated the effects of zofenopril and ramipril in a murine model of I/R injury (Figure 5). Mice were pretreated with vehicle, zofenopril (10 mg/kg PO), or ramipril (3 mg/kg PO) 8 hours before 45 minutes of ischemia and 24 hours of reperfusion (Figure 5A). Four mice from the vehicle group (n=16) and 5 mice from the zofenopril‐treated group (n=14) were excluded from INF size analysis because of technical difficulties during the staining procedure. The myocardial AAR/LV was similar between zofenopril‐ and vehicle‐ (63.4±0.9 versus 64.5±3.7%) treated groups (Figure 5B), indicating consistency in the extent of injury induced by surgical induction of I/R injury in mice. After 8 hours of zofenopril pretreatment, there was a significant reduction in myocardial INF/AAR (33.6±3.72 versus 47.6±4.5%, *P*<0.05) at 24 hours of reperfusion compared with vehicle (Figure 5B). Although it did not reach significance, there was a trend for reduction in INF per LV with zofenopril pretreatment compared with vehicle (21.3±2.4 versus 31.1±3.8%, *P*=0.059) (Figure 5B). The reduction in INF/AAR in the zofenopril‐treated mice was accompanied by a significant reduction in circulating cTn‐I levels at 4 hours of reperfusion compared with vehicle (Figure 5C; 3.4±1.3  versus 10.7±2.1 ng/mL, *P*<0.01). These findings indicate that zofenopril, when administered as a single dose 8 hours before myocardial ischemia, exerts cardioprotective effect on ischemia‐induced cardiac injury. As observed in the zofenopril‐ and vehicle‐treated groups, consistency in the amount of cardiac tissue subjected to ischemic injury at time of surgical coronary artery occlusion (AAR/LV) was similar between the ramipril‐ and vehicle‐ (61.0±2.5% versus 60.2±1.6%) treated groups (Figure 5D). Ramipril pretreatment resulted in a significantly smaller INF/AAR (27.1±3.3 versus 38.0±4.2%, *P*<0.05) and INF/LV (15.9±2.1 versus 22.9±2.5%, *P*<0.05) compared with vehicle (Figure 5D). At 4 hours of reperfusion, circulating cTn‐I levels were also significantly reduced in the ramipril‐treated group compared with vehicle (6.7±0.83  versus 9.2±0.82 ng/mL, *P*<0.05; Figure 5E).

### Zofenopril Reduces Acute Myocardial I/R Injury in Swine

**Figure 6:**
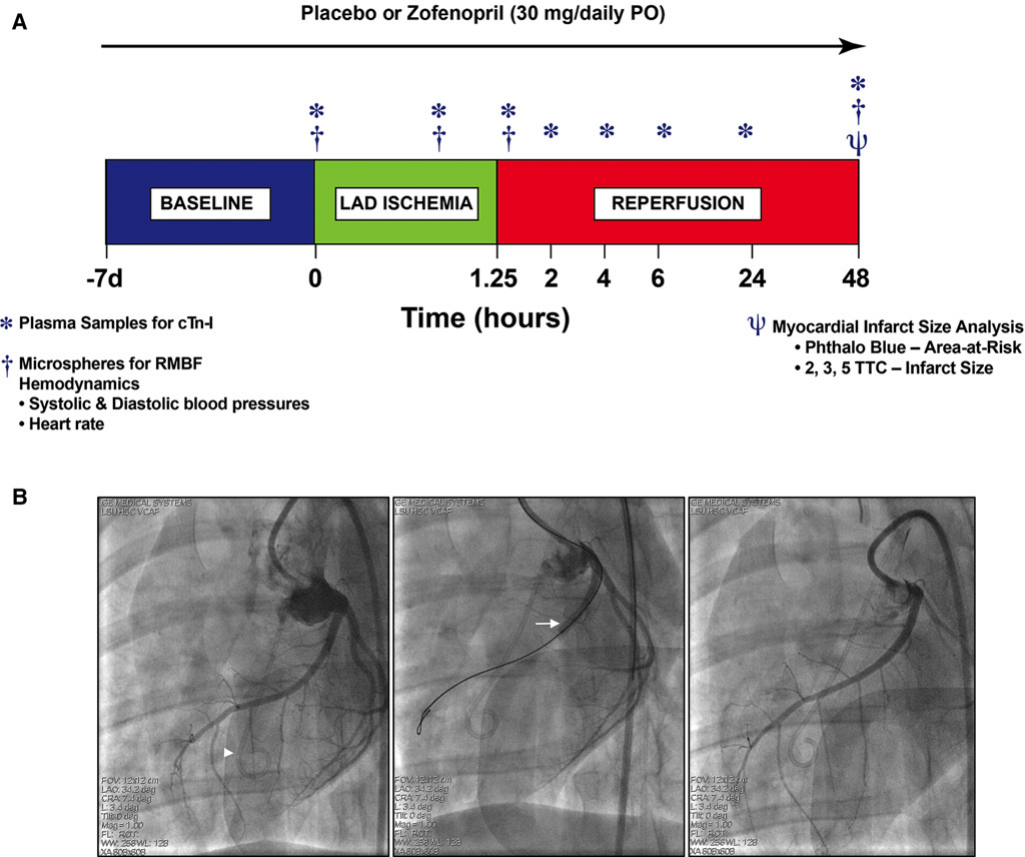
Experimental protocol for in vivo myocardial ischemia/reperfusion (I/R) injury in swine. A, Female Yucatan pigs were subjected to 75 minutes (1.25 hours) of myocardial ischemia by occluding left anterior descending coronary artery (LAD) via a balloon catheter placement and 48 hours of reperfusion. Zofenopril (30 mg/daily PO) or placebo therapy was initiated at 7 days prior I/R injury and continued for 2 days after ischemia. At baseline, 60 minutes of LAD occlusion, 15 minutes of reperfusion, and 48 hours of reperfusion, microspheres labeled with samarium, europium, lutetium, or lanthanum were injected to measure regional myocardial blood flow (RMBF). At baseline, 60 minutes of ischemia, and 15 minutes, 2, 4, 6, 24, and 48 hours of reperfusion, plasma samples were collected for measurement of cardiac troponin‐I release. At day 2 of reperfusion, heart tissue was collected for infarct size determination. Baseline plasma samples were used for assessment of circulating levels of H_2_S, sulfane sulfur, NO_2_^−^, and *S*‐nitrosothiols (RXNO). B, Angiographic left anterior oblique caudal images of the LAD at baseline (left), during 75 minutes occlusion (middle), and at 15 minutes reperfusion (right). Intracoronary occlusion was achieved by inflation of an angioplasty balloon catheter (arrow) deployed in the proximal LAD distal to the first anterior septal branch. Proximal LAD deployment of the balloon produced an area‐at‐risk (AAR) region ≈45% of the left ventricular (LV) mass. The AAR was distributed primarily in the anterior LV free wall and includes a portion of the anterior septum. For measurement of regional blood flow, microspheres were injected into the LV cavity through a pigtail catheter (arrowhead).

**Figure 7:**
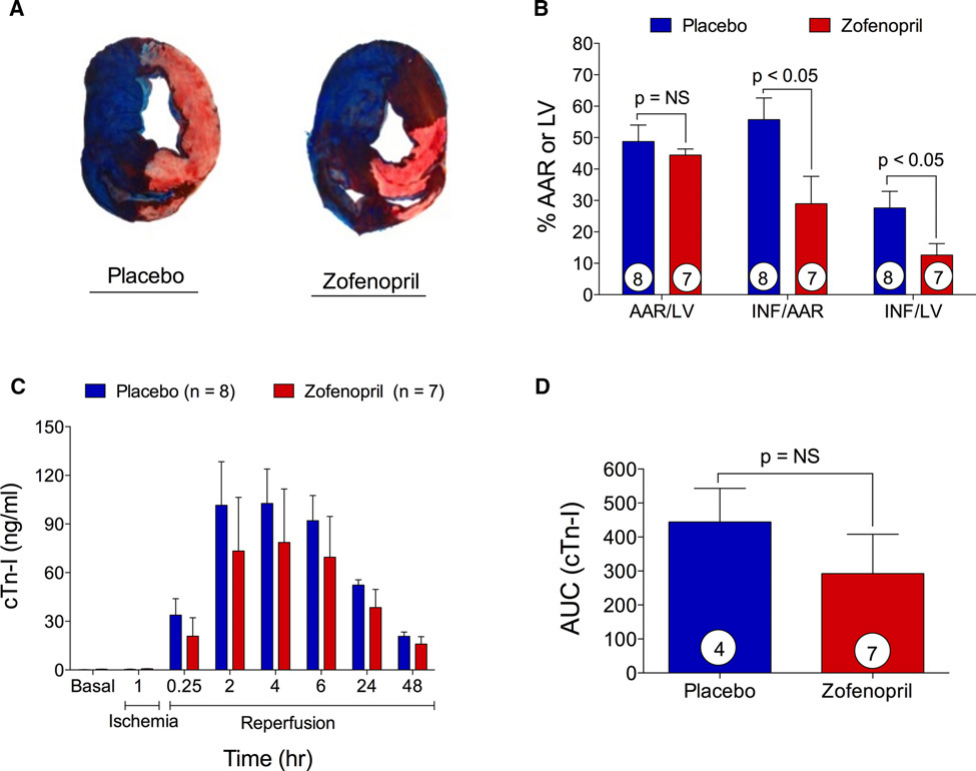
Reduction of infarct size by zofenopril pretreatment in a swine model of myocardial ischemia/reperfusion (I/R). A, Representative mid‐ventricular heart slices from placebo‐ and zofenopril‐treated animals. Area‐at‐risk (AAR) per left ventricle (LV) and infarct (INF) per AAR and LV were determined with a dual stain technique. There was no difference in AAR/LV between placebo‐ and zofenopril‐treated groups. B, Zofenopril pretreatment significantly reduced INF/AAR and INF/LV compared with placebo. C and D, Plasma levels of cTn‐I measured at time 0, 60 minutes of ischemia, and 15 minutes and 2, 4, 6, 24, and 48 hours of reperfusion and cumulative release of cTn‐I. Zofenopril reduced cTn‐I release during reperfusion with no significant extent. Results are expressed as mean±SEM. Number in the circle inside the bar denotes the number of animals used per group.

Building on the positive results in mice demonstrating zofenopril's ability to increase circulating and tissue H_2_S and ${\text{NO}}_{2}^{-}$ levels, increased eNOS tissue activation, and reducted INF size and troponin I release, we then evaluated whether zofenopril pretreatment would be cardioprotective in a clinically relevant, large animal model of I/R injury. As illustrated in the experimental protocol (Figure 6A), I/R injury was induced in pigs on day 0 after pretreatment for 1 week (starting on day −7) with placebo or zofenopril (30 mg/daily PO). Figure 6B are illustrative angiographic cine images of the LAD at baseline (*left*), during 75 minutes of occlusion (*middle*) and at 15 minutes of reperfusion (*right*). Table 1 contains all data with respect to group assignment from the swine I/R injury experiments. Of the total 17 pigs enrolled in the study, 2 pigs were excluded from the zofenopril group because of LAD dissection during balloon occlusion and fatal ventricular arrhythmias unresponsive to cardioversion. One additional pig had 7 days of zofenopril pretreatment and was killed before I/R injury for collection of plasma and cardiac tissue for biochemical analysis. There were no significant differences between placebo‐ and zofenopril‐treated groups with respect to age, body weight, number of defibrillations, heart rate, rate‐pressure product, and mean arterial blood pressure during occlusion and after reperfusion. Illustrative photographs of mid‐ventricular slices from placebo‐ and zofenopril‐treated animals demonstrate the degree of infarction (Figure 7A). Placebo‐ and zofenopril‐treated animals displayed a similar AAR/LV, indicating consistency between groups with respect to balloon placement during LAD occlusion. After pretreatment with zofenopril for 7 days, there was a significant reduction in myocardial INF/AAR (28.9±8.7 versus 55.7±6.9%, *P*<0.05) and INF/LV (12.6±3.7 versus 27.6±5.3%, *P*<0.05) compared with placebo (Figure 7B). Serial measurements of circulating cTn‐I levels were determined at 0, 60 minutes of LAD occlusion, 15 minutes, and 2, 4, 6, 24, and 48 hours of reperfusion (Figure 7C). As expected, there was a time‐dependent release of cTn‐I in both treatment groups, with the highest circulating levels measured at 2 to 6 hours into reperfusion. Surprisingly, in the swine model of I/R injury, the significant reduction in INF size after pretreatment with zofenopril did not correlate with a reduction in cTn‐I levels at any of the time points tested or analyzed as total cumulative cTn‐I release (Figure 7D). These results indicate that 1 week pretreatment with zofenopril before the myocardial ischemia event and its continued administration during reperfusion leads to a reduction in myocardial INF size without a concomitant reduction of circulating cTn‐I levels.

**Table 1:** Data From the Pig Myocardial Ischemia/Reperfusion Injury Model

	Placebo Mean±SE (n=8)	Zofenopril Mean±SE (n=9)	*P* Value
Exclusions (No. of pigs excluded/No. of pigs assigned)	1/8 (13%)	2/9 (22%)	0.313
Age, d	287±12 (8)	284±10 (9)	0.849
Body weight, kg	42.4±2.2 (8)	40.9±1.7 (9)	0.593
No. of defibrillations	3.4±2.3 (8)	4.9±1.8 (7)	0.313
Heart rate, bpm			
Baseline	84.6±6.3 (8)	78.3±7.4 (9)	0.266
60‐min occlusion	79.1±3.0 (8)	78.6±7.2 (7)	0.471
15‐min reperfusion	88.6±4.6 (8)	83.9±7.1 (7)	0.288
48‐h reperfusion	92.8±4.0 (8)	93.0±8.0 (7)	0.489
Systolic blood pressure, mm Hg			
Baseline	117.4±8.4 (8)	131.8±4.7 (9)	0.073
60‐min occlusion	113.8±4.1 (8)	102.9±8.0 (7)	0.115
15‐min reperfusion	115.9±9.7 (8)	104.9±8.3 (7)	0.206
48‐h reperfusion	96.6±8.9 (8)	89.4±4.1 (7)	0.248
Diastolic blood pressure, mm Hg			
Baseline	79.3±4.6 (8)	84.7±7.6 (9)	0.282
60‐min occlusion	95.0±4.7 (8)	80.1±9.0 (7)	0.076
15‐min reperfusion	90.6±8.2 (8)	71.7±6.4 (7)	0.049
48‐h reperfusion	64.4±6.9 (8)	62.6±4.4 (7)	0.417
Mean arterial pressure, mm Hg			
Baseline	97.6±5.2 (8)	112.9±4.4 (9)	0.019
60‐min occlusion	103.5±4.5 (8)	91.4±8.4 (7)	0.106
15‐min reperfusion	102.5±9.3 (8)	86.9±6.5 (7)	0.102
48‐h reperfusion	74.5±7.3 (8)	75.9±4.7 (7)	0.266
Rate‐pressure product			
Baseline	9806±784 (8)	10 432±1249 (9)	0.343
60‐min occlusion	9040±609 (8)	8298±1308 (7)	0.300
15‐min reperfusion	10 545±1467 (8)	8957±1349 (7)	0.222
48‐h reperfusion	8948±831 (8)	8282±786 (7)	0.285

### Zofenopril Enhances Subendocardial Blood Flow at Reperfusion

**Figure 8:**
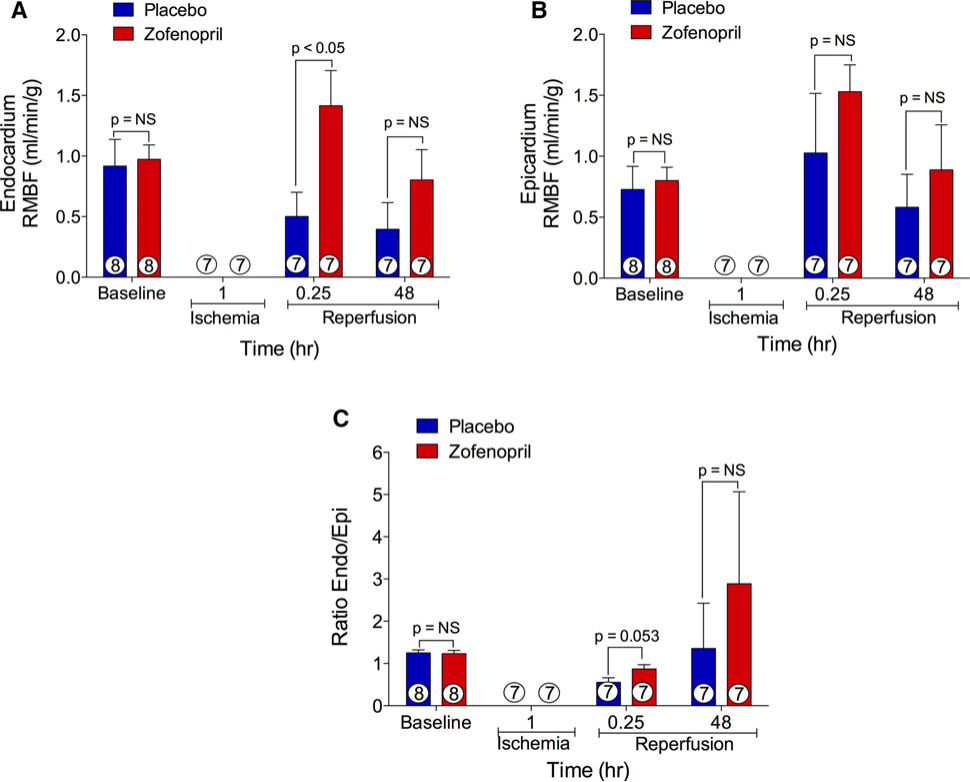
Regional myocardial blood flow. A, Endocardial myocardium blood flow, (B) epicardial myocardium blood flow, and (C) the ratio endocardial/epicardial blood flow in the ischemic zone at baseline, 60 minutes of left anterior descending coronary artery occlusion, and 15 minutes and 48 hours of reperfusion. Zofenopril resulted in a significantly greater endocardial blood flow at 15 minutes of reperfusion compared with placebo. Number inside the bar denotes the number of animals used per group.

Myocardial perfusion of the endocardial and epicardial ischemic and nonischemic regions was determined by using stable‐isotope neutron‐activated microspheres, and RMBF results are presented in Figure 8. In the zofenopril‐pretreated hearts, RMBF was significantly increased in the endocardial ischemic zone measured at 15 minutes after reperfusion (1.4±0.3  versus 0.5±0.2 mL/min per gram; *P*<0.05) compared with placebo (Figure 8A). Moreover, the RBMF endocardial:epicardial ratio in the ischemic zone at 15 minutes into reperfusion trended higher in the zofenopril‐pretreatment group (0.9±0.1  versus 0.6±0.1 mL/min per gram, *P*=0.053) (Figure 8C) compared with placebo. RMBF in the epicardial ischemic zone was not different between study groups at any time‐point (Figure 8B). There were no differences in RMBF detected in epicardial and endocardial RMBF nonischemic zones between groups (Table 2). Taken together, the increase in blood flow observed in the endocardial ischemic region in the pigs pretreated with zofenopril may contribute to the observed reduction in myocardial INF size.

**Table 2:** Nonischemic Regional Myocardial Blood Flow

	Placebo Mean±SE (n=8)	Zofenopril Mean±SE (n=9)	*P* Value
Baseline RMBF, mL/min per gram			
Nonischemic zone endocardium	0.847±0.18 (8)	1.106±0.19 (8)	0.247
Nonischemic zone epicardium	0.702±0.13 (8)	0.896±0.14 (8)	0.228
Nonischemic zone endocardium/epicardium ratio	1.254±0.10 (8)	1.218±0.10 (8)	0.351
60‐min occlusion RMBF, mL/min per gram			
Nonischemic zone endocardium	1.008±0.33 (7)	0.662±0.14 (7)	0.083
Nonischemic zone epicardium	0.921±0.27 (7)	0.782±0.24 (7)	0.231
Nonischemic zone endocardium/epicardium ratio	1.183±0.10 (7)	0.945±0.15 (7)	0.161
15‐min reperfusion RMBF, mL/min per gram			
Nonischemic zone endocardium	1.014±0.33 (7)	0.762±0.15 (7)	0.125
Nonischemic zone epicardium	1.357±0.53 (7)	0.802±0.14 (7)	0.088
Nonischemic zone endocardium/epicardium ratio	0.883±0.12 (7)	0.959±0.11 (7)	0.368
48‐h reperfusion RMBF, mL/min per gram			
Nonischemic zone endocardium	1.175±0.26 (7)	1.267±0.25 (7)	0.486
Nonischemic zone epicardium	1.199±0.30 (7)	1.272±0.49 (7)	0.488
Nonischemic zone endocardium/epicardium ratio	1.069±0.12 (7)	1.324±0.32 (7)	0.250

RMBF indicates regional myocardial blood flow.

### Effects of Zofenopril on H_2_S, Sulfane Sulfur, ${\text{NO}}_{2}^{-}$, and RXNO Levels

**Figure 9:**
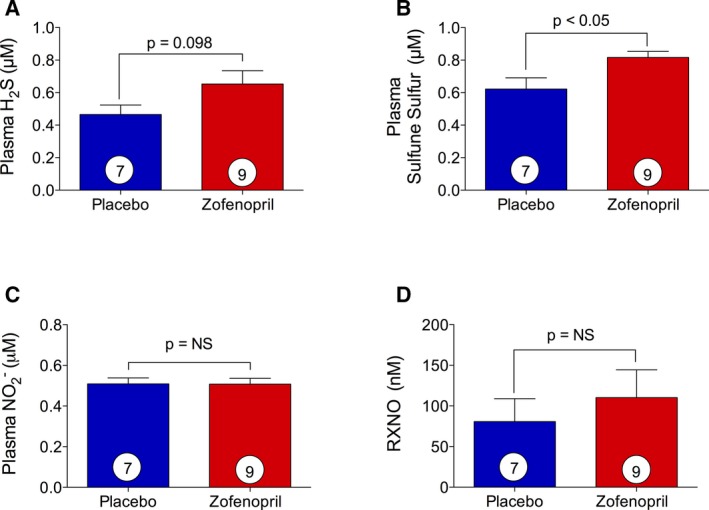
Effect of zofenopril on circulating H_2_S, NO_2_^−^, sulfane sulfur and *S*‐nitrosothiols (RXNO). At day 7 of placebo or zofenopril treatment of swine, plasma samples were harvested prior surgical procedure of myocardial ischemia reperfusion (I/R) to assess H_2_S, sulfane sulfur, NO, and RXNO levels. A, Zofenopril therapy did not significantly increase plasma H_2_S availability. B, Data for levels of sulfane sulfur demonstrate a significant increase in zofenopril‐treated animals compared with placebo. C, Zofenopril treatment did not alter circulating nitrite levels. D, Zofenopril treatment for 7 days did not alter circulating RXNO levels. Results are expressed as mean±SEM. Number in the circle inside the bar denotes the number of animals used per group.

Since a single administration of zofenopril in healthy mice increased circulating H_2_S and ${\text{NO}}_{2}^{-}$ levels at 8 hours after treatment, we next determined whether prolonged treatment with zofenopril increased circulating H_2_S levels in swine. Animals were pretreated with once‐daily placebo or zofenopril (30 mg) for 7 days before I/R injury with continued once‐daily placebo or zofenopril during the 48 hours of reperfusion (Figure 6A). Following 1 week of placebo or zofenopril pretreatment and before balloon occlusion, plasma samples were collected to measure circulating levels of H_2_S, sulfane sulfur, NO metabolites, ${\text{NO}}_{2}^{-}$, and RXNO. There were no differences in circulating H_2_S levels after 1 week of zofenopril pretreatment compared with placebo (Figure 9A, *P*=0.098). Sulfane sulfur, or acid‐labile sulfur,^47^ is considered to be a storage reservoir of H_2_S^48^ that forms when H_2_S binds to plasma proteins. Zofenopril pretreatment for 7 days resulted in a significant increase in plasma sulfane sulfur levels (0.82±0.04  versus 0.62±0.07 μmol/L, *P*<0.05) compared with placebo (Figure 9B). There were no differences in plasma ${\text{NO}}_{2}^{-}$ and RXNO levels between the zofenopril‐ and placebo‐pretreated groups (Figure 9C and 9D). These data suggest that prolonged zofenopril treatment results in a significant accumulation of H_2_S that is selectively bound to plasma proteins and not circulating freely in healthy swine. We can further speculate that during the acute onset of myocardial ischemia, one mechanism by which zofenopril exerts cardioprotection is the rapid release of H_2_S from the circulating storage reservoir.

## Discussion

Myocardial infarction is responsible for the vast majority of morbidity and mortality^49^ associated with cardiovascular disease. Currently, there are a number of ongoing trials testing preconditioning and postconditioning treatments that aim to diminish tissue damage during myocardial I/R. Preconditioning studies examining various pharmacological agents, such as mitochondrial K^+^_ATP_ channels openers,^50^ NO, or nitroxyl (HNO) donors^51^, ^52^ have been shown to reduce myocardial ROS formation^53^, ^54^, ^55^ and myocardial damage under ischemic conditions. Recently, exogenously delivered H_2_S and/or enhancement of the endogenous H_2_S signaling pathway has been shown to limit cardiac injury acutely after I/R injury.^56^, ^57^, ^58^ Indeed, it has been demonstrated that H_2_S has effects on INF sparing after I/R injury but is also able to exert potent antioxidant,^59^, ^60^ antiapoptotic,^61^ anti‐inflammatory,^62^ and proangiogenic^36^ effects under various stressors. In addition, the biological profile of H_2_S is similar to that of NO, and these 2 endogenous gaseous signaling molecules may affect one another with respect to their production and cell signaling molecular pathways. Similar to NO, H_2_S evokes vasorelaxing effects and eNOS inhibition or endothelium removal attenuates H_2_S‐induced vasorelaxation in rat aortic tissue.^63^, ^64^ In addition, our recent work shows that H_2_S donors activate eNOS and augments NO bioavailability in CSE KO mice.^39^ Furthermore treatment with H_2_S donors or modulation of the endogenous production of H_2_S through the cardiac specific overexpression of CSE protects against AMI and heart failure by attenuating oxidative stress, inhibiting apoptosis, and reducing inflammation.^59^

Currently, ACEIs are widely used for the treatment of hypertension and play a pivotal role in the management of morbidity and mortality associated with myocardial ischemia and heart failure. Many double‐blinded randomized trials have demonstrated the benefits of ACEIs on survival in heart failure patients with LV systolic dysfunction. Among all ACEIs, zofenopril is different in that it is a sulfhydryl‐containing, highly lipophilic compound that possesses ancillary and cardioprotective properties. It is zofenopril's unique pharmacology that makes it a desirable therapy for many cardiovascular diseases, including myocardial infarction and heart failure.^19^, ^65^ Following extensive evidence of its efficacy and safety derived from randomized controlled studies (ie, SMILE 1–4), zofenopril is currently indicated for treatment of patients initiated within the first 24 hours after AMI.^22^ Further, the efficacy of zofenopril has been reported to be superior to that of ramipril, in terms of prevention of major cardiovascular outcomes.^7^ In addition, a number of preclinical and clinical studies have demonstrated that zofenopril exerts additional effects beyond that of ACE inhibition^6^, ^19^, ^66^ with a recent study reporting that zofenopril‐mediated improvement of peripheral vascular function involves H_2_S signaling and is independent of ACE blockade.^23^

Previous studies have investigated the effects of zofenopril in experimental models of I/R injury and have shown that the protection afforded by zofenopril is partially abolished by BK receptor antagonist.^67^ Further, zofenopril improves cardiac contractile force and reduces lactate dehydrogenase release during reperfusion and the INF size in isolated rat hearts subjected to global I/R injury.^68^ Another study, in which zofenopril effect on recovery of contractile function after a short period of ischemia in dogs was compared with a non–sulfhydryl‐containing ACEI, enalaprilat, revealed that enalaprilat attenuates postischemic dysfunction, at least in part by a prostaglandin‐mediated signaling, whereas beneficial effects of zofenopril are mainly associated to the antioxidant properties of its sulfhydryl moiety and preservation of protein thiols at the end of ischemia.^69^ However, it has also been reported that zofenopril stimulates active calcium uptake through sarcoplasmic reticulum cycling in the cardiomyocytes, which could account for improvements in myocardial contractility after I/R.^70^ In addition, improvement of postischemic LV function, increase in coronary blood flow, reduction of myocardial cell injury, creatine kinase release, lipid peroxidation^71^ and myocardial norepinephrine release^72^ all account for zofenopril‐mediated cardioprotection. Last but not least, it has also been demonstrated in a swine model of I/R that 2 days of zofenopril pretreatment significantly reduces the pressure‐rate product, an index of myocardial oxygen demand, and decreases the peak efflux of epinephrine, norepinephrine, and adenosine catabolites in the coronary venous effluent.^73^ Therefore, zofenopril clearly exerts a number of beneficial effects beyond ACE inhibition whose precise mechanisms remain still under investigation.

We examined the bioavailability of H_2_S and NO in the circulatory system and cardiac tissue after a single dose of zofenopril in mice or prolonged therapy in swine. Further, we investigated whether short‐ or long‐term preconditioning by zofenopril administration could prevent and thereby limit the cardiac damage after reperfusion using both murine and swine models of in vivo I/R injury. Moreover, we have also determined the effects of zofenopril treatment on oxidative stress before the occurrence of the ischemic injury.

**Figure 10:**
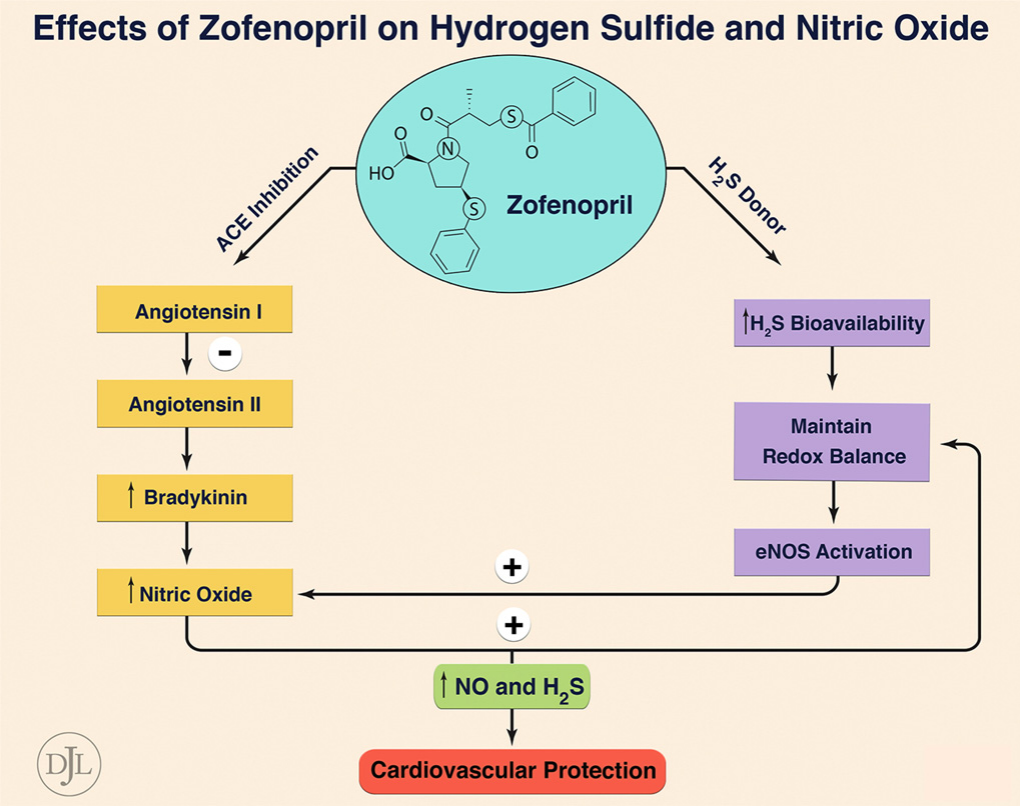
Effect of zofenopril on H_2_S and NO bioavailability. By inhibiting myocardial angiotensin converting enzyme (ACE) activity, zofenopril reduces the generation of angiotensin II and increases levels of bradykinin (BK). BK, through stimulation of endothelial B_2_ receptors, promotes the release of NO, prostacyclin, and endothelium‐derived hyperpolarizing factor (EDHF), which in turn leads to cardioprotection. On the other hand, zofenopril, by releasing H_2_S, enhances tissue antioxidant defense and promotes eNOS activation, leading to increased levels of NO. Therefore, ACE inhibition, H_2_S, and NO account for zofenopril‐mediated cardioprotective effects.

Our findings clearly demonstrate that zofenopril potentiates H_2_S and NO bioavailability. In particular, zofenopril administered as a single dose in mice increases the levels of both H_2_S and ${\text{NO}}_{2}^{-}$ in myocardial and plasma tissue. These data are supported by the swine study, in which we did not observe increased levels of free H_2_S but did find higher levels of sulfane sulfur after 7 days of zofenopril treatment. Conversely, the augmented levels of NO after zofenopril treatment are the result of an increased phosphorylation of eNOS at the Ser1177 site, which promotes eNOS activation. We believe that eNOS activation may be induced by direct release of H_2_S by zofenopril or by inhibition of BK metabolism because of the ACEI activity. BK through stimulation of endothelial B_2_ receptors promotes the release of vasodilatory agents like prostacyclin and endothelium‐derived hyperpolarizing factor and eNOS activation leading to increased NO bioavailability (Figure 10). The enhanced H_2_S signaling, alongside the inhibition of angiotensin II formation and BK metabolism, could represent a further explanation of the additional beneficial effects afforded by zofenopril.

Further, preconditioning with zofenopril in both mice and swine models of in vivo I/R injury resulted in a significant reduction in myocardial INF size, reductions in cTn‐I release during reperfusion in mice, and a greater coronary perfusion of ischemic zone early into reperfusion in swine. Thus, zofenopril exerts cardioprotective effects beyond ACE inhibition by augmenting H_2_S and NO before ischemia and limiting the myocardial damage after I/R. The presence of a sulfhydryl group in the molecular structure of zofenopril may account for the release of H_2_S, which in turn may explain in part how zofenopril protects the ischemic myocardium. During hypoxia and I/R conditions, ROS are the main factor of cardiac tissue damage. Therefore, we examined whether zofenopril preconditioning could enhance tissue antioxidant defense preventing ROS formation and following ischemic injury during reperfusion. In our study, we observed that treatment with zofenopril upregulated the antioxidant enzymes Trx‐1, GPx‐1, and SOD‐1, suggesting an increase in antioxidant defenses before ischemia that mitigate myocardial reperfusion injury. Therefore, our findings demonstrate that zofenopril‐mediated release of H_2_S and NO can scavenge ROS directly and/or indirectly via upregulation of antioxidant defense, resulting in the prevention of ischemia‐induced cardiac damage. In conclusion, our data suggest that zofenopril exerts cardioprotective actions via NO and H_2_S signaling that extend beyond ACE inhibition. However, additional studies are required to more fully elucidate the precise mechanisms involved in the non‐ACEI effects of zofenopril in the setting of myocardial infarction and heart failure.

## Sources of Funding

This study was supported by a Menarini Industrie Farmaceutiche Riunite grant. In addition, this work was supported by grants from the National Heart, Lung, and Blood Institute (National Institutes of Health; 1R01 HL092141 [Dr Lefer], 1R01 HL093579 [Dr Lefer], 1U24 HL 094373 [Dr Lefer], 1P20 HL113452 [Dr Lefer]) and an Ochsner Translational Medical Research Institute Grant. We are also grateful for the generous funding from the Louisiana State University Medical School Alumni Association.

## Disclosures

Dr Evangelista is an employee of Menarini Richerche Spa, which is part of the Menarini Group, the maker of zofenopril. The other authors have nothing to disclose.
